# Noise levels in general pediatric facilities: A health risk for the staff?

**DOI:** 10.1371/journal.pone.0213722

**Published:** 2019-03-13

**Authors:** Peter Voitl, Christian Sebelefsky, Christoph Mayrhofer, Astrid Woditschka, Verena Schneeberger

**Affiliations:** 1 First Vienna Pediatric Medical Center, Vienna, Austria; 2 Sigmund Freud Private University, Vienna, Austria; 3 Department of Pediatrics and Adolescent Medicine, Medical University of Vienna, Vienna, Austria; University College London, UNITED KINGDOM

## Abstract

This study was initiated to investigate noise levels in general pediatric facilities. Although occupational noise limits of 85dBA for L_Aeq,8h_ (daily noise exposure) and 140dBC for L_Cpeak_ (peak sound level) have proven to prevent hearing loss, even low levels of continuous noise (45dBA and above) can cause adverse health effects (ISO = International Organization for Standardization, A = Austrian VOLV). The sound level measurements of L_Aeq_ (equivalent sound level) and L_Cpeak_ were conducted with a decibel meter in the examination rooms (EXR) and waiting rooms (WR) of 10 general pediatric practices and outpatient clinics in the city of Vienna, Austria. L_Aeq,8h_ was calculated from L_Aeq_, and independent variables with a potential influence on noise levels were also examined. In EXR, the random sample consisted of 5 to 11 measuring periods per facility (mean: 7.1 ± 1.9) with a total duration between 43.85 and 98.45 min. (total: 10:19:04). With L_Aeq_ ranging from 67.2 to 80.2dBA, specific recommended limits were exceeded considerably (ISO: 45dBA; A: 50dBA). In WR, the random sample comprised 5 to 18 measurements per facility (mean: 13.7 ± 5.0) with a total duration ranging from 25 to 90 min. (total: 11:25:00). The values for L_Aeq_ were between 60.6dBA and 67.0dBA. All of these significantly exceeded recommended limits of 55dBA (ISO) and 5 out of 10 exceeded 65dBA (A). L_Cpeak_ reached 116.1dBC in WR and 114.1dBC in EXR. The highest calculated daily noise exposure of pediatricians (L_Aeq,8h_) was 79dBA. Although no significantly increased risk for hearing loss can be concluded from our findings, it must be assumed that noise levels in general pediatrics have the potential to cause stress and associated health issues. Further research is necessary to foster the recognition of noise-related health impairments of pediatric staff as occupational diseases.

## Introduction

### Background

#### Noise levels at medical departments

It has been demonstrated over the last decades that healthcare personnel are exposed to considerable noise [1–15]. Several studies have addressed noise levels and exposure at specialized non-pediatric medical departments [1–5]. Pediatric patients represent a potential source of remarkable noise, which is why many investigations have been conducted at PICUs (pediatric intensive care unit) [6–10], and other facilities treating young patients [11–15]. Ratnapalan et al. [13] investigated noise levels in a pediatric emergency department and showed an average noise level (Leq) of 68.73 dB for a 24-hour period and a maximum-recorded noise level of 110 dB. To our knowledge, noise levels and exposure of the staff at general pediatric facilities have not been addressed so far. This circumstance provided the starting point for our research efforts, particularly, as many procedures in general pediatrics (e.g. vaccinations) involve significant noise levels.

#### Noise at the workplace–regulations and recommendations

The most common epidemiological measure of daily noise exposure at the workplace is L_Aeq,8h_ (daily personal noise exposure) [16]. An exposure of 85 dBA (L_Aeq,8h_) or 140 dBC (L_Cpeak_ = peak sound level) and above is widely accepted to represent a clear risk for impaired hearing [16–23]. It should be noted, that these limits represent an “acceptable” risk, as opposed to being risk-free. Accordingly, exposure to even markedly lower noise levels in the range of background noise can cause serious physical and psychological problems, ranging from annoyance to undesirable physiological effects [24–27]. In order to avoid these, more restrictive noise limits have been proposed [17, 23, 28, 29]. Depending on the applied standard and the field of work, even background noise levels of 45 dBA (ISO 11690–1:1996(en)) at the workplace are desirable [28].

Hence, low background noise is necessary to ensure good communication between pediatrician and parents of pediatric patients in healthcare settings. When background noise levels increase above normally low levels they become distracting and annoying. This distracting or annoying noise is considered separately from noise with significant potential to cause hearing loss.

See Table 1 for a comprehensive summary of international regulations and recommendations regarding noise at the workplace.

**Table 1:** Regulations for noise exposure and recommended limits for specific noise levels at the workplace.

Regulations for noise exposure and recommended limits for specific noise levels at the workplace		Quantity [unit]					
		L_Aeq,8h_ [dBA]	L_Cpeak_ [dBC]	L_Aeq_ [dBA]			
				Routine office work	Intellectual tasks	Desired for work	Causing annoyance at the workplace
**Organization /** **regulation / directive**	ISO 1999:2013(en) [18] / ISO 11690–1:1996(en) [28]	85	140	55	45		
	NIOSH [19]	85	140				
	OSHA [20, 30]	90	140				
	ACGIH [21]	85					
	ANSI [22]		140				
	AAP [29]					50	60
	European Directive 2003/10/EC [31]	87	140				
	Austrian VOLV [23]	85	137	65	50		
	VDI Richtlinie 2058 Blatt 3 (DIN, VDI) [17]			70	55		

#### Noise-induced hearing loss (NIHL)

Noise-induced hearing loss is one of the most prevalent occupational diseases worldwide. In the US and Europe, it is the most reported occupational disease overall [32]. This also applies to Austria in particular, accounting for more than one half of all work-related diseases (c.f., approx. one third in Europe), ahead of skin and respiratory problems [33]. Most of the cases are reported in the manufacturing and construction sector although cases occur throughout all occupational fields [32, 33].

#### Other adverse health effects of occupational noise exposure

Although hearing damage has been the main concern of safety regulations ever since, other physical and psychological effects may not be neglected. Exposure to noise levels exceeding specific recommended limits may have further undesirable impacts. These include but are not limited to tinnitus, temporary threshold shift, elevated blood pressure, reduced performance, sleeping difficulties, annoyance, and stress [24]. Even at a daily noise exposure below 85 dBA, there are provable psychological (e.g., anger, strain or nervousness) and physical responses (e.g., increased blood pressure or excretion of magnesium), which in further consequence may engender long-term disorders of regulation mechanisms [27]. According to selected study results, at least one third (35–45%) of office workers find noise levels above 55 dBA extremely irritating [25].

A high complexity level of tasks is likely to affect the efficiency of workers [27] It also determines how sensitive they will react to disturbances like noise [27]. The confidence of a person in their ability is furthermore essential, which makes untrained personnel more vulnerable [27]. In addition, intermittent noise tends to be more disruptive than continuous noise [26]. This especially applies when the periods of noise can hardly be predicted or controlled [26]. It has also been shown that in noisy environments antisocial behavior is more likely to be observed [26].

As the relationship between noise exposure of pediatric staff and adverse auditory and non-auditory health effects is not scientifically proven and, therefore, these are not recognized as occupational diseases, the estimated number of unknown cases might be considerably high.

### Objectives

The primary objective of this investigation was to measure noise levels at general pediatric facilities. In addition, the noise exposure of pediatric staff was of special interest. Secondary objectives were to ascertain variables that influence noise levels in general pediatric settings.

## Methods

### Study design, study sites, and participants

This cross-sectional observational study was conducted at 10 general pediatric facilities (nine pediatric practices and one outpatient clinic) throughout the city of Vienna, Austria (Table 2). Sound levels were metered in 10 different examination rooms (one at each facility if several existed) and 10 different waiting rooms (one at each facility). The measurements were conducted during summer from June to July 2014.

**Table 2:** Numbers and names of participating general pediatric facilities.

Number of facility	Name of facility
1	First Vienna Pediatric Medical Center (general pediatric outpatient clinic)
2	Dr. Birgit Hofmann-Ehrhart (pediatric practice)
3	Dr. Yelena Tiedt-Oberbauer (pediatric practice)
4	Dr. Angelika Thaler (pediatric practice)
5	Dr. Andreas Doczy (pediatric practice)
6	Dr. Helmuth Howanietz (pediatric practice)
7	Dr. Johann Sommer (pediatric practice)
8	Dr. Nicole Grois (pediatric practice)
9	Dr. Sylvia Stein-Krumholz (pediatric practice)
10	Dr. Georg Maiwald (pediatric practice)

Primarily, the sound levels in examination rooms and waiting rooms were measured using a sound level meter. In addition, sex and age of the examined pediatric patients were gathered, numbers of parents and children present in the waiting rooms were counted and the time needed for the examination was measured.

All thirty-seven resident pediatricians with a contract with the statutory health insurance (SHI) listed on the homepage of the Vienna Medical Association (Ärztekammer für Wien) were contacted by e-mail and asked to take part in the study. Nine of these doctors responded and agreed to participate. In a second step, further non-SHI-accredited resident pediatricians were contacted until one of these agreed to participate. Non-SHI-accredited doctors do not have a contract with public health insurance companies and bills are not directly covered by these. Patients, therefore, are obliged to pay the provided services on their own and a certain amount of the costs can be recovered from their insurance company. The number of ten pediatric facilities were chosen to obtain a reliable sociodemographic sample throughout different districts and ethnical groups. The participating pediatric facilities are situated in 9 of the 23 Districts of Vienna (1^st^, 2^nd^, 3^rd^, 8^th^, 9^th^, 10^th^, 21^st^, 22^nd^, 23^rd^).

All parents or legal guardians (aged 18 and above) and pediatric patients (aged 0–17), as well as all kinds of consultations and diseases, could be included in the investigation. Parents and legal guardians with insufficient German language skills were excluded.

### Ethical approval

The study has been approved by the Ethics Committee of the Medical University of Vienna (protocol no. 2190/2013). Participating families and the completed questionnaires remained anonymous at any time, and participation in the study was voluntary. Informed consent was obtained from all individual participants included in the study. All parents and pediatric patients (or their siblings) at the age of 11 and above, as well as all staff members being present in waiting rooms and examination rooms, had to assent to participate in written form.

### Data collection

#### Sound level meter and sound parameters

All measurements were performed with a Brüel & Kjær 2250 Light sound level meter. The sound level was metered in examination rooms and waiting rooms. Before starting and after finishing the measurement, the sound level meter was calibrated. The sound level meter was set to display L_Cpeak,1s_ (peak sound level during the latest second), L_Cpeak_ (peak sound level), and L_Aeq_ (equivalent sound level). L_Cpeak_ and L_Aeq_ were recorded till the end of each measurement. L_Cpeak,1s_ was only read out to determine actual peak sound level events during ongoing measurements. Reasons for impulsive noise events were annotated immediately. In addition, the quietest consultation of all examination rooms and the quietest 5-minute-interval in waiting rooms were determined (L_Aeqmin_). This was also done for the noisiest measurements (L_Aeqmax_). In all respective cases, the average L_Aeq_ of measurements in examination rooms and waiting rooms was calculated using the standard formula for equivalent sound pressure levels (logarithmic average). The arithmetic mean of equivalent sound levels was only calculated to determine the average L_Aeq_ of certain age groups of pediatric patients.

#### Calculation of daily noise exposure

In order to estimate the actual daily noise exposure of pediatric staff, the measured L_Aeq_ values were converted to occupation group specific L_Aeq,8h_ values. This was done using the highest L_Aeq_ of all facilities, measured in examination rooms and waiting rooms respectively, as well as the average L_Aeq_ of all examination rooms and waiting rooms. Hence, four L_Aeq_ values were used for conversion in total. An online calculating tool, the “Daily Noise exposure calculator” from the Health and Safety Executive (HSE) homepage [34], served for this purpose. In order to obtain representative values for an 8-hour working day (L_Aeq,8h_), four location-specific L_Aeq_ values, including the proportional time of exposure, were entered into the tool as part of each of the four calculations. The proportional time periods of exposure were estimated by protocolling an 8-hour working day of three doctors and doctor’s receptionists of facility 1 each and averaging the results for the respective occupation group. The location-specific L_Aeq_ values included:

- the measured values for L_Aeq_ in examination rooms (highest and average) with 6.75 hours of exposure for pediatricians and 0.25 hours for doctor’s receptionists
- the measured values for L_Aeq_ in waiting rooms (highest and average) with 0.25 hours of exposure for pediatricians and 6.75 hours for doctor’s receptionists
- approximate values for L_Aeq_ in common rooms (50 dBA) with 0.75 hours of exposure for both pediatricians and doctor’s receptionists
- approximate values for L_Aeq_ in restrooms (45 dBA) with 0.25 hours of exposure for both pediatricians and doctor’s receptionists

When calculating L_Aeq,8h_ from the highest L_Aeq_ in examination rooms, the L_Aeq_ obtained in the respective waiting room was used. The same applies vice versa to the highest L_Aeq_ in waiting rooms. For calculation of L_Aeq,8h_ related to the average L_Aeq_ only the respective two values for examination rooms and waiting rooms were used with the appropriate time of exposure.

#### Examination rooms

All of the participating pediatric facilities have at least two and a maximum of five examination rooms. In order not to interfere with the work flow during usual opening hours the measurements took place at a single examination room.

While not being attended by a doctor, parents and pediatric patients (and their siblings) at the age of 11 or above were asked if they consent to the sound level measurement during the succeeding examination. In addition, they were informed about content and purpose of the study, the necessary procedures, and the voluntariness of their participation. If parents and children were willing to participate in the study, they had to read and sign a consent form. If they declined to participate, the measurement was abstained from. Before starting the measurement, the patient’s sex and date of birth (month and year) were indicated by parents or patients themselves. This information was annotated anonymously and subsequently transferred to an Excel file.

The measurement was commenced as soon as any staff member (nurse, resident or consultant) was present in the examination room, even if the patient was first prepared for the actual examination (e.g. weighing, measuring length or head circumference). One member of the research team not otherwise engaged held the device close to the doctor during the entire measurement period. In case the measurement was started without the doctor being present in the examination room, the investigator remained seated at the doctor’s desk until the doctor entered. This course of action was chosen as doctors of all facilities generally have to take care of medical documentation before attending patients. In case the nurse had finished preparatory tasks before the doctor entered the examination room, the measurement was paused until then. As soon as the doctor had left the room, the measurement was stopped. The investigator did not interact with the other personnel at any time.

In seven facilities the measurements took place between 8 am and 12 am and in the three remaining facilities between 1 pm and 5 pm. At each study site noise levels were metered from Monday through Friday in a single session. One measurement period refers to one consultation of a family. In case parents attended with several children needing medical attention, only one measurement was performed and the sound level meter recorded continuously.

At eight out of ten study sites weighing and measuring height was done in the examination room. This does not apply to facility 4 and 6, where pediatric patients were weighed and measured before entering the examination room. For these insitutions, the duration of time needed for one consultation consequently does not include this time period.

#### Waiting rooms

In each waiting room, the sound level meter was placed in an appropriate position to allow representative measurements. The dedicated member of the research team selected this position which was close to a doctor’s receptionist in all facilities. Prior to starting the measurements, accompanying parents were informed about content and purpose of the study and necessary procedures were explained. They were furthermore made aware of the voluntariness of their participation. Families attending during an ongoing measurement were informed accordingly. Two minutes and thirty seconds after the measurement had been started, all children and parents in the room were counted (excluding staff). The measurements were done at 5-minute intervals and should preferably be repeated 18 times, resulting in a total planned observation period of 90 minutes per facility. This approach allowed taking into account variations of noise levels within a working day. Whenever the waiting room was empty for more than ten minutes (two measuring periods) the measurement was stopped. In each facility, the measurements were conducted in one session from Monday through Friday between 8 am and 12 am.

### Statistical methods

The data collection and descriptive analysis were carried out using Microsoft Excel (Version 15.32). IBM SPSS Statistics (Version 22.0) was used for statistical testing. Variables comprised the sound levels in examination rooms and waiting rooms (L_Aeq_, L_Cpeak_, L_Aeqmax_, L_Aeqmin_), the sex and age of the examined pediatric patients, the total number of pediatric patients and parents present in the waiting room, as well as the time needed for the examination. Each variable was evaluated descriptively and statistical relationships between variables related to noise levels and each of the other variables were examined. For this purpose, the significance level was set to 0.05. The number of consultations was only evaluated descriptively (mean and standard deviation).

The Spearman correlation and Spearman’s rank correlation coefficient were used to examine the statistical relationship between sound levels and the number of people in waiting rooms. The T-test was used to identify statistically significant differences between the sound levels produced by boys and girls. In addition, sound levels (L_Aeq_ and L_Cpeak_) were evaluated with respect to specific age groups.

## Results

### Examination rooms

**Fig 1:**
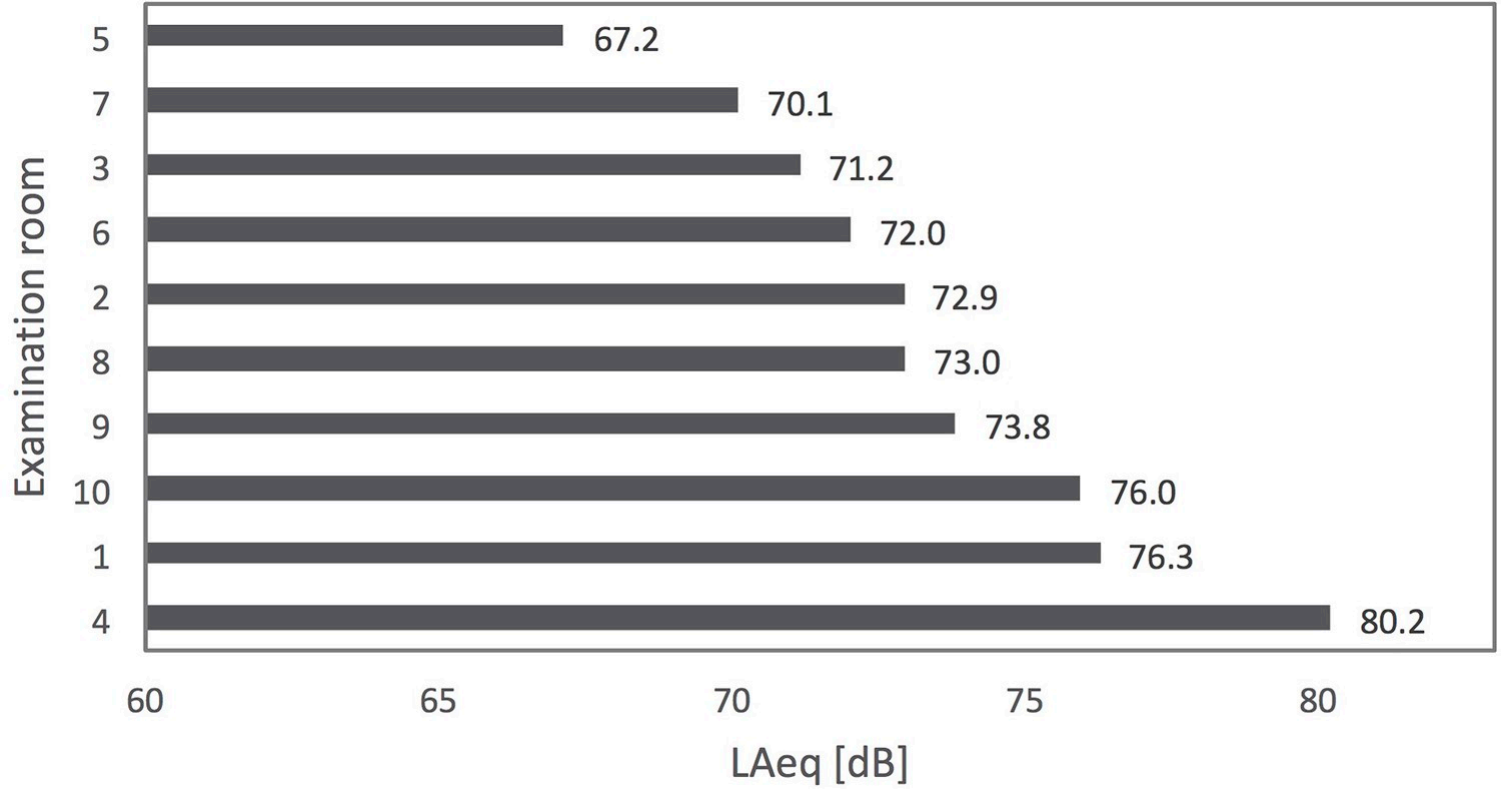
Equivalent sound levels (L_Aeq_) in examination rooms.

**Fig 2:**
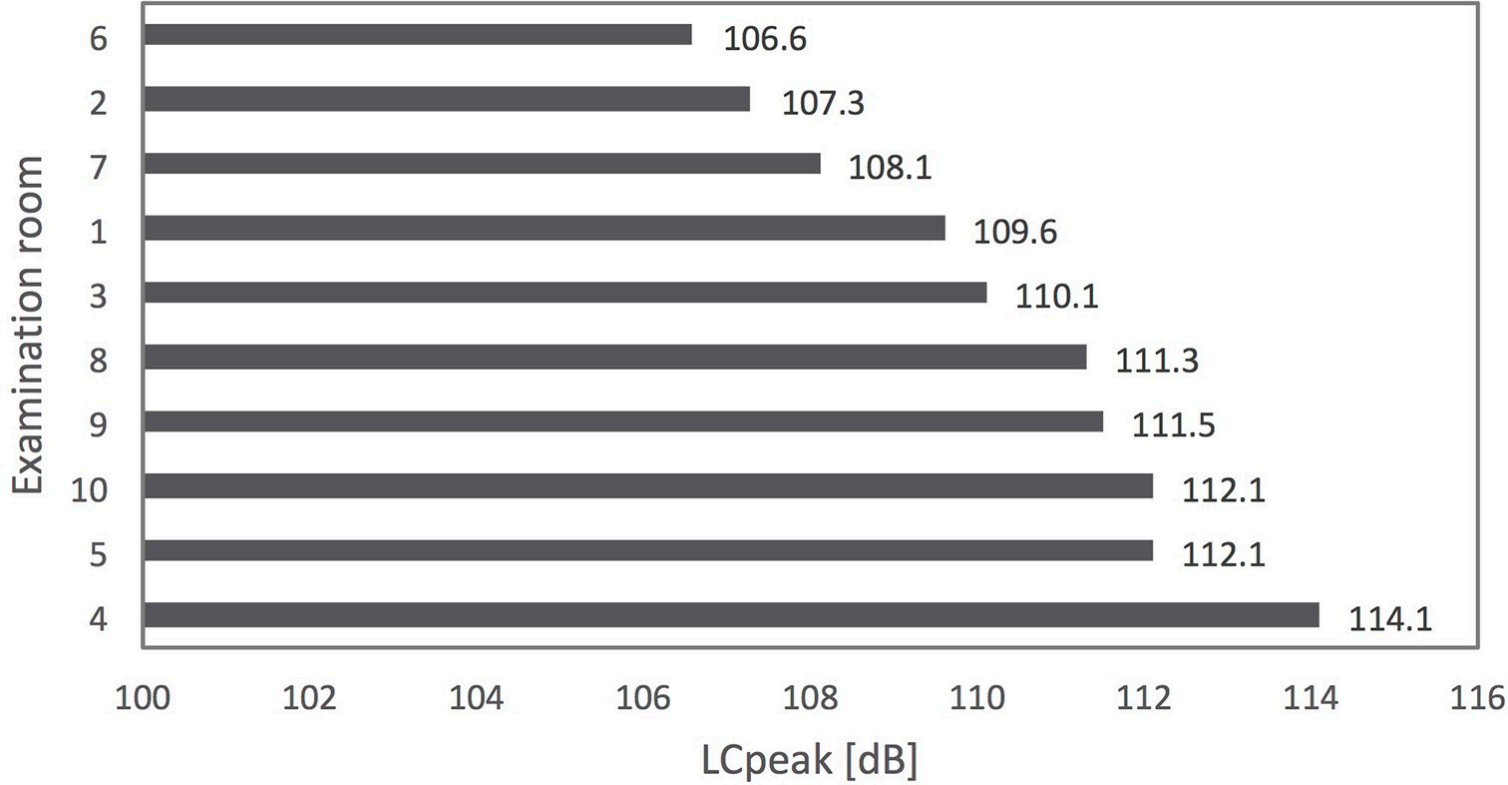
Peak sound levels (L_Cpeak_) in examination rooms.

The values of the energy equivalent sound pressure level (L_Aeq_) ranged from 67.2 dBA to 80.2 dBA (Fig 1). These are the sound levels of the total measuring period of each of the study sites. The highest calculated L_Aeq,8h_, affecting doctors and referring to the highest measured L_Aeq_ of 80.2 dBA (facility 4), was 79 dBA. The minimum sound levels (L_Aeqmin_) of each of the measuring periods (quietest consultation) ranged from 56.1 dBA to 65.9 dBA. The maximum sound levels (L_Aeqmax_) were between 74.1 dBA and 90.3 dBA (noisiest consultation). The peak sound levels (L_Cpeak_) of all facilities ranged from 106.6 dBC to 114.1 dBC (Table 3 and Fig 2). The average L_Aeq_ of all consultations and facilities was 74.5 dBA. The calculated average L_Aeq,8h_ of all facilities affecting doctors was 74 dBA.

**Table 3:** Sound levels in examination rooms with duration of examinations and measurements.

Examination room	1	2	3	4	5	6	7	8	9	10
L_Aeq_ [dB]	76.3	72.9	71.2	80.2	67.2	72.0	70.1	73.0	73.8	76.0
L_Aeqmin_ [dB]	57.1	63.8	59.3	60.3	58.9	63.3	56.1	60.5	65.9	61.1
L_Aeqmax_ [dB]	84.1	80.2	76.2	90.3	74.3	78.2	74.1	75.5	81.1	89.2
L_Cpeak_ [dB]	109.6	107.3	110.1	114.1	112.1	106.6	108.1	111.3	111.5	112.1
Median duration of examination [min.]	11.12	9.33	20.20	4.10	7.56	4.10	9.26	9.31	10.71	5.67
Total duration of measurement [min.]	66.35	57.62	81.48	48.93	51.45	50.28	55.57	65.08	98.45	43.85

The median examination of all facilities lasted 8.20 minutes. For the shortest examination, only one minute was needed, the longest took 25.35 minutes. The median duration of examinations of each facility ranged from 4.10 minutes to 20.20 minutes per consultation. The sound level measurements were conducted during 71 consultations (5 to 11 per facility, mean: 7.1 ± 1.9). The total duration of measurements ranged from 43.85 to 98.45 minutes per facility and was 10 hours, 19 minutes and 4 seconds in total.

**Fig 3:**
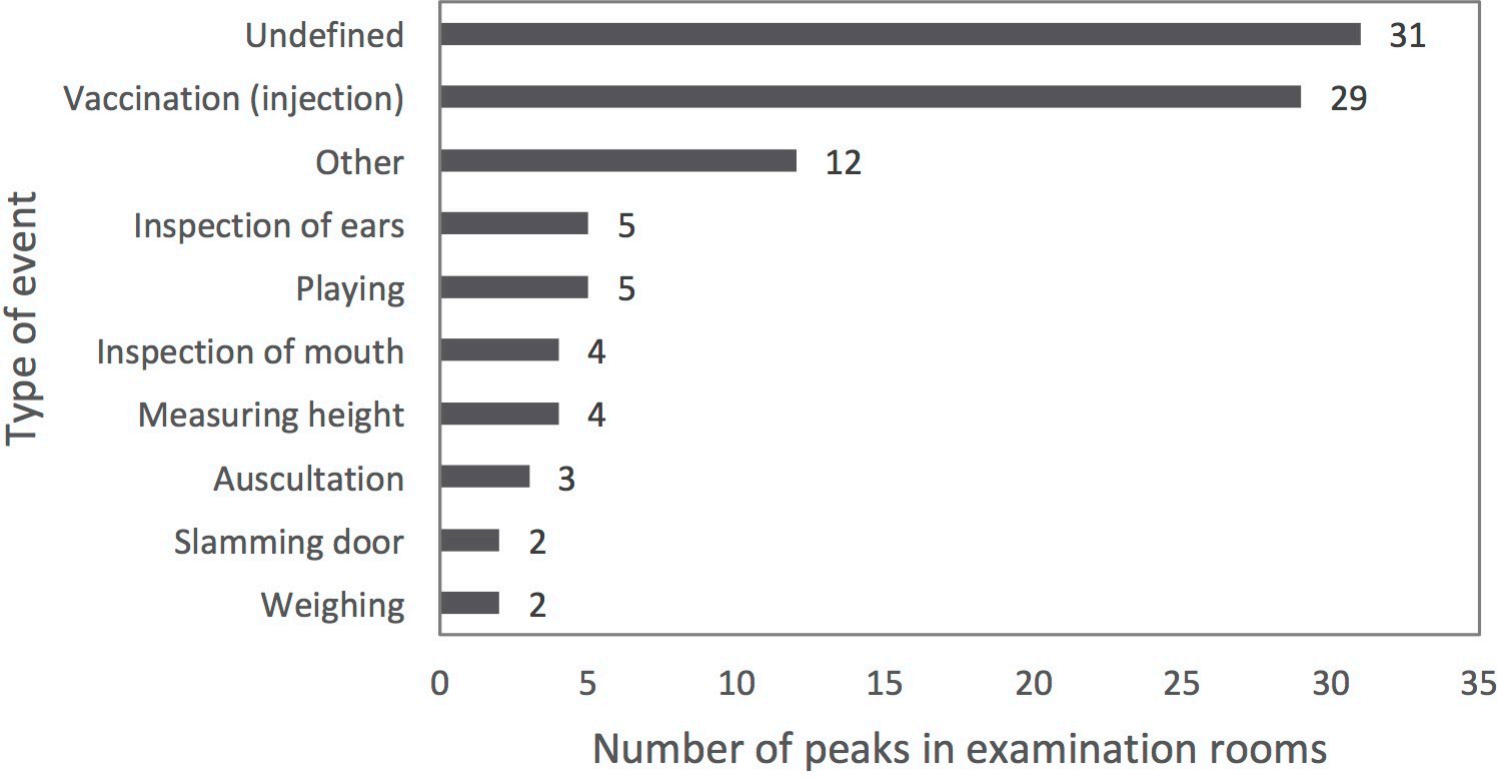
Types of peak sound level events and their frequencies.

A total number of 97 peak sound level (L_Cpeak_) events were gathered, of which the reason could be identified in 66 cases. The most common among these were vaccinations with 29 occurrences. These were followed by other (non-itemized) events (n = 12), inspections of the ears and playing children (both n = 5), and inspections of the mouth and measuring height (both n = 4) (Fig 3).

Pediatric patients were between 1 month and 15 years of age, the median age was 16 months, and the average age was 32.4 months. Out of 85 examined children 52 (61.2%) were boys and 33 (38.8%) were girls.

**Fig 4:**
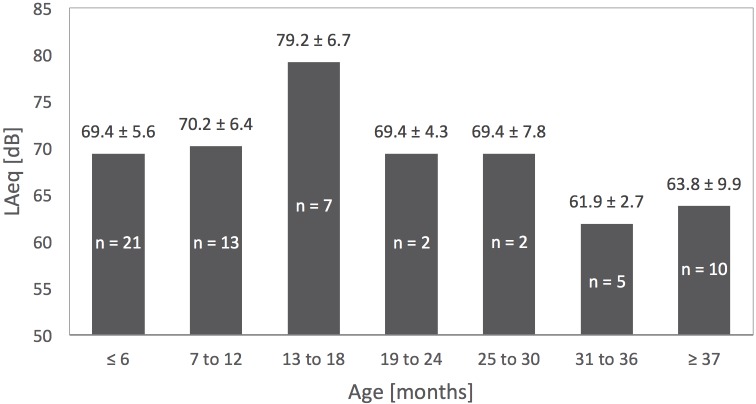
Age groups of pediatric patients and their specific average L_Aeq_ (examination rooms).

The highest average L_Aeq_ (79.2 dBA) was observed in the age group 13 to 18 months (Fig 4). The pertaining values for each group were calculated using the arithmetic mean, after dividing the children into seven age groups. Consultations with more than one child present in the examination room were excluded. This descriptive analysis was not done for L_Cpeak_, as too many peak sound levels were not caused by pediatric patients or the corresponding source could not be identified.

### Waiting rooms

**Fig 5:**
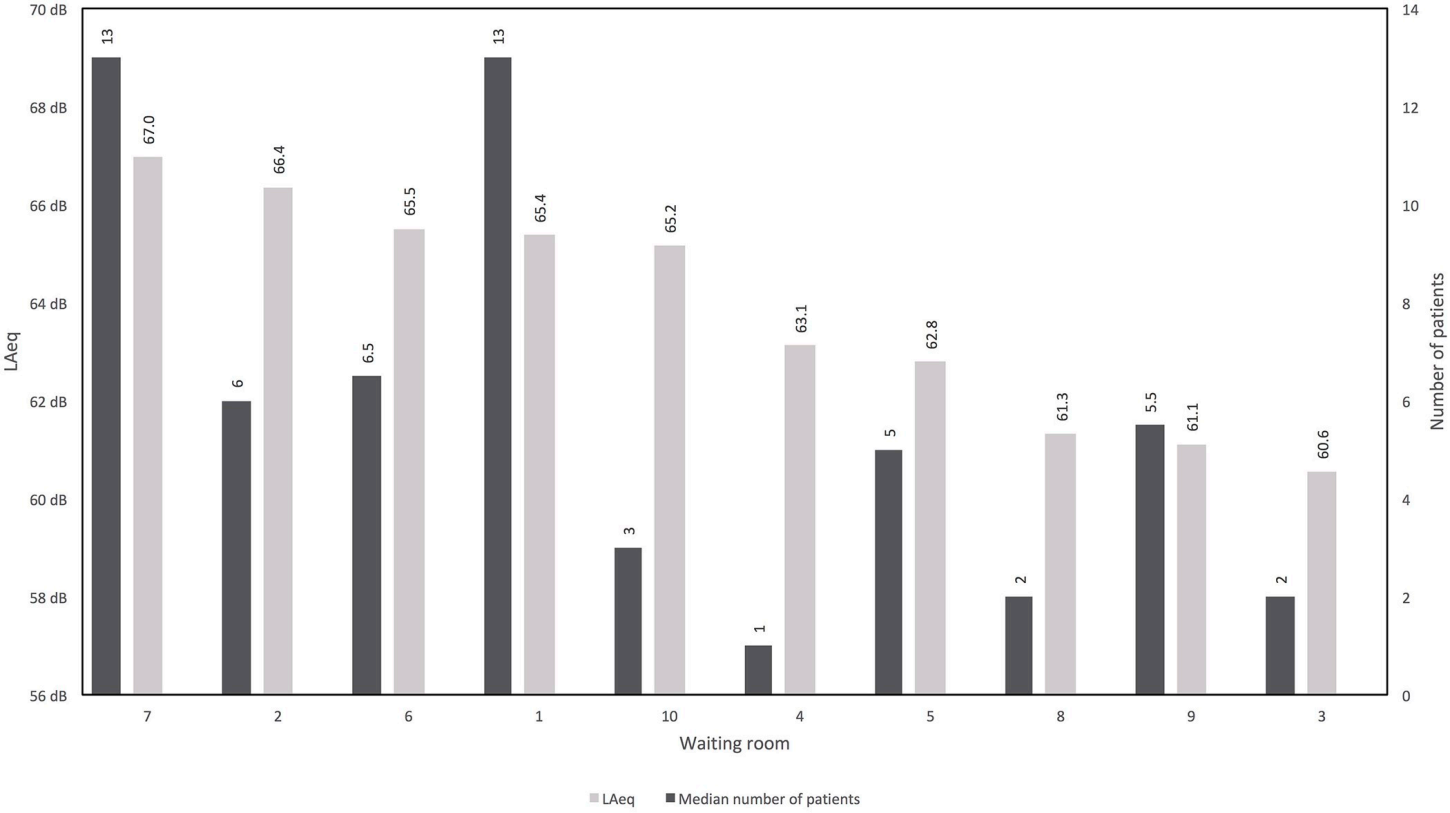
Equivalent sound levels (L_Aeq_) and median number of pediatric patients in waiting rooms.

The values of L_Aeq_ in the waiting rooms of all pediatric facilities ranged from 60.6 dBA to 67.0 dBA (Fig 5). These are the sound levels of the total measuring period of each of the study sites. The highest calculated L_Aeq,8h_, affecting doctor’s receptionists and referring to the highest measured L_Aeq_ of 67.0 dBA (facility 7), was 65 dBA. The minimum sound levels (L_Aeqmin_) were between 43.6 dBA and 61.4 dBA (quietest measuring interval). The maximum sound levels (L_Aeqmax_) ranged from 62.7 dBA to 75.3 dBA (noisiest measuring interval). L_Cpeak_ of all facilities ranged from 103.7 dBC to 116.1 dBC (Table 4). The average L_Aeq_ of all measuring periods and facilities was 65.1 dBA. The calculated average L_Aeq,8h_ of all facilities affecting doctor’s receptionists was 65 dBA.

**Table 4:** Sound levels in waiting rooms with numbers of children and adults and duration of measurements.

Waiting room	1	2	3	4	5	6	7	8	9	10
L_Aeq_ [dB]	65.4	66.6	60.6	63.1	62.8	65.5	67.0	61.3	61.1	65.2
L_Aeqmin_ [dB]	59.8	58.1	56.2	59.6	49.6	55.3	58.1	43.6	56.0	61.4
L_Aeqmax_ [dB]	69.1	74.4	62.7	68.4	68.2	75	75.3	70.0	70.0	67.3
L_Cpeak_ [dB]	112.2	109.1	104.2	108.1	110.1	108.1	107.5	103.7	103.9	116.1
Median no. of children	6.5	2.5	1	0	3	3	6	1	3	1
Median no. of adults	7	3.5	1	1	2	3	7	1	2	2
Duration of measurement [min.]	90	90	30	55	65	80	90	70	90	25

In six out of ten facilities the waiting room was empty for more than ten minutes (two measuring periods) during the measurement, which was therefore stopped. Consequently, instead of 180 measurements (5-minute intervals) only 137 were conducted (5 to 18 per facility, mean: 13.7 ± 5.0), resulting in a total measurement time of 11 hours and 25 minutes with measuring periods ranging from 25 to 90 min. per facility.

The number of persons being present in the waiting rooms at the same time ranged from 0 to 14 adults and from 0 to 13 children. The total number of people being present at the same time ranged from 0 to 27. The median number of persons ranged from 0 to 6.5 children and from 1 to 7 adults (Fig 5). The median total number of persons ranged from 1 to 13.

During 137 measurement periods, there were 30 reasons accounting for peak sound levels (L_Cpeak_). The most prominent reason with 12 peaks were children playing with toys, followed by slamming doors with 11 peaks. During 107 measurement periods, no specific reason for peak sound level events could be identified.

### Statistical relationships

There is no statistical difference between the sex of children regarding the energy equivalent sound level L_Aeq_ (t = -0.008, p = 0.994) and the peak sound level L_Cpeak_ (t = -0.127, p = 0.900).

There is a highly significant statistical connection between the number of people in waiting rooms and the energy equivalent sound level L_Aeq_ (Spearman ρ = 0.485, p < 0.01). More people in the room caused higher levels of L_Aeq_. This also applies to L_Cpeak_ (Spearman ρ = 0.348, p < 0.01). More people in the room caused higher sound levels for L_Cpeak_.

## Discussion

### Discussion of results

This study represents the first attempt to investigate noise levels and noise exposure in general pediatric facilities, which explains the mainly descriptive approach. None of the approximate values for L_Aeq,8h_ affecting pediatricians and doctor’s receptionists exceeded the common noise exposure limit of 85 dBA [16–19, 21]. The highest observed daily exposure of pediatricians was 79 dBA (facility 4), and the average daily exposure of all pediatricians was 74 dBA. The highest daily exposure affecting receptionists was 65 dBA (facility 7), and the average daily exposure of all receptionists was also 65 dBA. The exposure limit of 80 dBA, which is the lower exposure action value (European Directive 2003/10/EC) [31] and also specifies noise exposure above release level (Austrian VOLV) [23], was not exceeded either.

None of the peak pressure levels (examination rooms: 106.6 to 114.1 dBC, waiting rooms: 103.7 to 116.1 dBC) exceeded common occupational noise limits (135, 137 or 140 dBC) [18–20, 22, 23, 31]. Altogether, these results provide some evidence for the conclusion that working at a general pediatric facility is not associated with a significantly increased risk of developing noise-induced hearing loss.

Nevertheless, daily exposure to even markedly lower noise levels can have adverse physical and psychological effects. The limit for background noise of 55 dBA while carrying out simple office work, recommended in ISO 11690–1:1996(en) [28], was exceeded in all waiting rooms. This also applies, when considering the limits of 50 dBA (desired for any work) and 60 dBA (causing annoyance) respectively, recommended by the American Academy of Pediatrics [29]. The limit of 65 dBA, recommended in the Austrian VOLV [23], was exceeded in 5 out of 10 waiting rooms. The average L_Aeq_ of all facilities (65.1 dBA) was above all three limits. Whereas the limit of 70 dBA (German VDI Richtlinie 2058 Blatt 3) [17] was not exceeded in waiting rooms. Altogether, these results still indicate that doctor’s receptionists, who often work in the waiting room or nearby, display an increased risk of becoming annoyed while at work and in further consequence developing stress-related health disorders.

In all examination rooms specific limits for background noise of 45 dBA (ISO 11690–1:1996(en)), 55 dBA (Austrian VOLV) [23] and 50 dBA (German VDI Richtlinie 2058 Blatt 3) [17], recommended when carrying out intellectual tasks, were exceeded considerably (lowest measured L_Aeq_: 67.2 dBA). This obviously also applies when using the noise limits of 50 dBA and 60 dBA, recommended by the American Academy of Pediatrics for any workplace [29]. All of this is also applicable to the average L_Aeq_ of 74.5 dBA. These results implicate that the professional activities of pediatricians involve an increased risk of developing annoyance and stress with all their diverse consequences. The results of Ratnapalan et al. [13] showed an average noise level (Leq) of 68.73 dB for a 24-hour period. Although obtained in a pediatric emergency department, these are also within the range of distracting and annoying noise levels, which corresponds to our findings (L_Aeq_ ranging from 67.2 to 80.2 dBA, average L_Aeq_: 74.5 dBA). An explanation for the higher values in our setting might be that frequent procedures in general pediatrics (e.g., vaccinations) involve significant noise levels. The maximum-recorded noise level of 110 dB in the study of Ratnapalan et al. [15] was within the range of our peak pressure levels in examination rooms (106.6 to 114.1 dBC).

The efficiency of a worker, and in our case the pediatrician, is more likely impacted if the complexity of a task is high [27]. As described in detail previously [26], this depends on the quantity of information that must be kept in mind, the proportion of required intellectual operations and tasks involving precise fine motor activity, as well as the extent to which the worker is responsible for consequences of mistakes. All of this distinctly applies to the work of pediatricians. The complexity also determines how sensitive a person will react to disturbances like noise [27]. In the unfavorable case, the number of mistakes increases, the completion of the task slows down, and consequently the efficiency decreases [27]. This is particularly crucial when treating patients.

When considering the adverse effects of noise on job performance, the individual experience and the individual judgment of a person‘s ability to perform the task also need to be taken into account [26]. Pediatric residents, for instance, are therefore at increased risk of displaying physical and psychological reactions. Furthermore, intermittent noise is far more disruptive than continuous noise, especially if it is unpredictable and uncontrollable [26]. This is obviously the case for noise produced by pediatric patients. It has been demonstrated before that less cooperative and antisocial behavior is more likely observed in noisy environments [26]. This is a particular problem for pediatric work, which, more than other disciplines, requires a well-collaborating and harmonic work environment.

Consequently, while administering vaccinations noise pollution might be a less severe problem in terms of treatment quality. Nonetheless, when making a diagnosis and developing individual therapy concepts for multimorbid and complex patients or familiarizing parents with complex issues, for instance, job performance might be affected considerably.

### Limitations

In our study, we decided to measure L_Aeq_ and estimate L_Aeq,8h_ from the obtained results. We chose this approach, as we expected to find values for L_Aeq_ within the range of distracting or annoying noise and no values for L_Aeq,8h_ above common noise exposure limits. In order to obtain most accurate results for L_Aeq,8h_, direct exposure measurements with a noise dosimeter, preferably over an 8-hour working day, would have been necessary. We measured L_Aeq_ during shorter periods of time as the main purpose of this study was to examine noise levels rather than noise exposure. Longer measuring periods presumably would have caused slightly lower values for L_Aeq_ and the calculated L_Aeq,8h_ respectively.

We did not carry out a power analysis to calculate sample size. In terms of measuring noise levels and estimating noise exposure by approximation, a sample size of 10 study sites with 71 consultations and 137 5-minute measurement periods in the waiting rooms was considered to be reasonable.

The primary objective of our investigation was to determine noise levels at general pediatric facilities. Since noise produced by pediatric patients contributes overproportionately to these, we did not conduct measurements without any occupants being present in examination rooms and waiting rooms. Nevertheless, particularly in those areas where noise is a disturbing or annoying influence rather than being a hearing health hazard, the convention is to measure the background noise under normal operating conditions without any occupants in the specific area. This would have been worth investigating additionally to determine background noise at the participating facilities.

The study was carried out during summer, resulting in a lower number of attending patients and supposedly in lower noise levels in the waiting rooms. Noise levels in the examination rooms were probably not affected, except for slight background noise.

The noise levels pediatric nurses, and other assistants carrying out auxiliary tasks, are exposed to, cannot be directly extrapolated from the noise levels we measured. This is due to the difference in work contents of nurses compared to pediatricians and doctor’s receptionists. Moreover, nurses often are not present in the room where the actual examination or treatment takes place. Nevertheless, they are still exposed to noise in examination rooms and waiting rooms, although their exposure is likely to exhibit a distinct profile.

Lastly, the validity of the results regarding sound levels produced by certain age groups is limited because of the small number of cases within the age groups (3 ≤ n ≤ 22).

## Conclusion

In conclusion, noise levels in general pediatric facilities definitely pose a health risk to the staff. More specifically, it must be assumed that noise exposure, especially of pediatricians, has the potential to cause disturbance, annoyance, and stress while at work, as well as further stress-related adverse health effects. Although our data do not provide evidence for an increased risk for hearing loss of pediatric staff, the observed noise pollution is still critical.

Future studies should focus on the effects of this specific noise environment, which include but are not limited to elevated blood pressure and other cardiovascular problems, reduced performance, sleeping difficulties, annoyance, and stress. Research projects in this field indubitably are of great importance when promoting the recognition of noise-related health impairments of pediatric staff as occupational diseases.

Future health prevention programs for pediatric staff should include noise assessments, audiometric monitoring of the personnel’s hearing, worker education, record keeping, and program evaluation. The organization of work, schedules, and rest periods also needs to be taken into account. The employer has a responsibility to assess and, if necessary, measure the levels of noise to which pediatric staff are exposed. As far as possible the risk factors must be reduced to a minimum.

## Supporting information

S1 File Complete data set. (XLSX)

## References

[1] Al-Dujaili M, Thomson WM, Meldrum R, Al-Ani AH. Noise levels in dental school clinics. The New Zealand dental journal. 2014;110(3):105–8. . 25265749

[2] Khademi G, Roudi M, Shah Farhat A, Shahabian M. Noise pollution in intensive care units and emergency wards. Iranian journal of otorhinolaryngology. 2011;23(65):141–8. 24303374 PMC3846184

[3] Kol E, Demircan A, Erdogan A, Gencer Z, Erengin H. The Effectiveness of Measures Aimed at Noise Reduction in an Intensive Care Unit. Workplace health & safety. 2015;63(12):539–45. 10.1177/2165079915607494 . 26493219

[4] Lester JD, Hsu S, Ahmad CS. Occupational hazards facing orthopedic surgeons. American journal of orthopedics. 2012;41(3):132–9. . 22530210

[5] Oliveira CR, Arenas GW. Occupational exposure to noise pollution in anesthesiology. Revista brasileira de anestesiologia. 2012;62(2):253–61. 10.1016/S0034-7094(12)70123-X . 22440380

[6] Bailey E, Timmons S. Noise levels in PICU: an evaluative study. Paediatric nursing. 2005;17(10):22–6. . 16372705 10.7748/paed.17.10.22.s21

[7] Carvalho WB, Pedreira ML, de Aguiar MA. Noise level in a pediatric intensive care unit. Jornal de pediatria. 2005;81(6):495–8. 10.2223/JPED.1424 . 16385369

[8] Milette IH, Carnevale FA. I'm trying to heal …noise levels in a pediatric intensive care unit. Dynamics. 2003;14(4):14–21. . 15453567

[9] Morrison WE, Haas EC, Shaffner DH, Garrett ES, Fackler JC. Noise, stress, and annoyance in a pediatric intensive care unit. Critical care medicine. 2003;31(1):113–9. 10.1097/01.CCM.0000037164.66392.AF . 12545003

[10] Watson J, Kinstler A, Vidonish WP 3rd, Wagner M, Lin L, Davis KG, et al Impact of Noise on Nurses in Pediatric Intensive Care Units. American journal of critical care: an official publication, American Association of Critical-Care Nurses. 2015;24(5):377–84. 10.4037/ajcc2015260 . 26330430

[11] Burk A, Neitzel RL. An exploratory study of noise exposures in educational and private dental clinics. Journal of occupational and environmental hygiene. 2016:1–29. 10.1080/15459624.2015.1072634 . 27077918 PMC4992430

[12] Calderon LE, Carney LD, Kavanagh KT. The Cry of the Child and its Relationship to Hearing Loss in Parental Guardians and Health Care Providers. Journal of evidence-informed social work. 2016;13(2):198–205. 10.1080/23761407.2015.1018031 . 25844672

[13] Jadid K, Klein U, Meinke D. Assessment of noise exposures in a pediatric dentistry residency clinic. Pediatric dentistry. 2011;33(4):343–8. . 21903003

[14] Marsh JP, Jellicoe P, Black B, Monson RC, Clark TA. Noise levels in adult and pediatric orthopedic cast clinics. American journal of orthopedics. 2011;40(7):E122–4. . 22013576

[15] Ratnapalan S, Cieslak P, Mizzi T, McEvoy J, Mounstephen W. Physicians' perceptions of background noise in a pediatric emergency department. Pediatric emergency care. 2011;27(9):826–33. 10.1097/PEC.0b013e31822c1357 . 21878828

[16] Concha-Barrientos M, Campbell-Lendrum D, Steenland K. Occupational noise: assessing the burden of disease from work-related hearing impairment at national and local levels. Geneva, World Health Organization, 2004. (WHO Environmental Burden of Disease Series, No. 9). Available from: http://www.who.int/quantifying_ehimpacts/publications/en/ebd9.pdf.

[17] Deutsche Gesetzliche Unfallversicherung (DGUV)—Fachbereich Holz und Metall—Berufsgenossenschaft Holz und Metall. DGUV-Information „Lärm-Stress”am Arbeitsplatz—Nicht das Innenohr betreffende, extra-aurale Lärm wirkungen 2013 [cited 19 June 2016]. Available from: http://www.dguv.de/medien/fb-holzundmetall/publikationen/infoblaetter/infobl_deutsch/018_LaermStressAmArbeitsplatz.pdf.

[18] International Organization for Standardization (ISO). ISO 1999:2013(en): Acoustics—Estimation of noise-induced hearing loss, (2013).

[19] National Institute of Occupational Safety and Health (NIOSH) (1998). Criteria for a Recommended Standard-Occupational Exposure to Noise—Revised Criteria 1998. DHHS (NIOSH) Pub. No.98-126. National Institute of Occupational Safety and Health, Cincinnati, Ohio [cited 19 June 2016]. Available from: http://www.cdc.gov/niosh/docs/98-126/pdfs/98-126.pdf.

[20] Occupational Safety and Health Administration (OSHA). Appendix II:A. General Industry Standard. Permissible Noise Exposures [cited 19 June 2016]. Available from: https://www.osha.gov/dts/osta/otm/noise/standards_more.html.

[21] Occupational Safety and Health Administration (OSHA). Section II: What standards limit and control noise exposure?—Other Standards and Guidance—The American Conference of Governmental Industrial Hygienists (ACGIH) [cited 19 June 2016]. Available from: https://www.osha.gov/dts/osta/otm/noise/standards.html.

[22] Occupational Safety and Health Administration (OSHA). Standards—Consensus Standards and Recommendations from other Professional Organizations—American National Standards Institute (ANSI) [cited 19 June 2016]. Available from: https://www.osha.gov/SLTC/noisehearingconservation/standards.html.

[23] Verordnung Lärm und Vibrationen (VOLV) [cited 19 June 2016]. Available from: https://www.jusline.at/Verordnung_Laerm_und_Vibrationen_(VOLV).html.

[24] Basner M, Babisch W, Davis A, Brink M, Clark C, Janssen S, et al Auditory and non-auditory effects of noise on health. Lancet. 2014;383(9925):1325–32. 10.1016/S0140-6736(13)61613-X 24183105 PMC3988259

[25] Passchier-Vermeer W, Passchier WF. Noise exposure and public health. Environ Health Perspect. 2000;108 Suppl 1:123–31. 10.1289/ehp.00108s1123 10698728 PMC1637786

[26] Suter AH. Communication and job performance in noise: a review. ASHA Monogr. 1992;(28):1–84. . 1364812

[27] World Health Organization (WHO). Occupational exposure to noise: Evaluation, prevention and control 1995 [cited 19 June 2016]. Available from: http://www.who.int/occupational_health/publications/noise.pdf?ua=1.

[28] International Organization for Standardization (ISO). ISO 11690–1:1996(en): Acoustics—Recommended practice for the design of low-noise workplaces containing machinery—Part 1: Noise control strategies, (1996).

[29] Noise: a hazard for the fetus and newborn. American Academy of Pediatrics. Committee on Environmental Health. Pediatrics. 1997;100(4):724–7. . 9836852

[30] Occupational Safety and Health Administration (OSHA). Occupational Safety and Health Standards—Occupational Health and Environmental Control (Part 1910, Subpart G, Standard no. 1910.95) [cited 19 June 2016]. Available from: https://www.osha.gov/pls/oshaweb/owadisp.show_document?p_table=STANDARDS&p_id=9735.

[31] DIRECTIVE 2003/10/EC OF THE EUROPEAN PARLIAMENT AND OF THE COUNCIL of 6 February 2003 on the minimum health and safety requirements regarding the exposure of workers to the risks arising from physical agents (noise) 2003 [cited 19 June 2016]. Available from: http://eur-lex.europa.eu/legal-content/EN/TXT/HTML/?uri=CELEX:02003L0010-20081211&from=EN.

[32] Stucken EZ, Hong RS. Noise-induced hearing loss: an occupational medicine perspective. Current opinion in otolaryngology & head and neck surgery. 2014;22(5):388–93. 10.1097/MOO.0000000000000079 . 25188429

[33] Allgemeine Unfallversicherungsanstalt (AUVA). Berufskrankheit—Häufigste Berufskrankheiten 2015 2016 [cited 19 June 2016]. Available from: http://www.auva.at/portal27/portal/auvaportal/content/contentWindow?contentid=10007.671002&action=2.

[34] Health and Safety Executive (HSE). Exposure calculators and ready-reckoners. Daily Noise exposure calculator. [cited 08 January 2017]. Available from: http://www.hse.gov.uk/noise/dailycalc.xls.

